# Understanding the role of hospice pharmacists: a qualitative study

**DOI:** 10.1007/s11096-021-01281-8

**Published:** 2021-06-13

**Authors:** Zoe Edwards, Emma Chapman, Simon Pini, Michael I. Bennett

**Affiliations:** University of Leeds, Leeds, UK

**Keywords:** Education, Hospice, Medical records, Palliative care, Patient care team, Pharmacists

## Abstract

*Background* Pharmacists are important members of multidisciplinary teams but, despite surveys of provision, the role of the hospice pharmacist is not well described. *Objective* To explore the role of the hospice pharmacist and identify barriers and facilitators to the role. *Setting* Hospices offering in-patient services caring for adults towards the end of life in one geographical area of northern England. *Method* Pharmacists providing services to hospices were invited to take part in qualitative semi-structured interviews asking about experience, patient contact, team working and barriers and facilitators to the role. These were recorded verbatim and data were analysed thematically using framework analysis. *Main outcome measure* The hospice pharmacist’s perceptions of their role and barriers and facilitators to it. *Results* Fifteen pharmacists took part. Two themes and ten subthemes were identified focused on tasks and communication. Practise was varied and time limited the quantity and depth of services carried out but was often spent navigating complex drug supply routes. Participants found methods of communication suited to the hours they spent in the hospice although communication of data was a barrier to effective clinical service provision. Participants identified the need for appropriate training and standards of practice for hospice pharmacists would enable better use of their skills. *Conclusion* Barriers to the role of hospice pharmacist include time, access to role specific training, access to clinical information and complex medicines supply chains. The role would benefit from definition to ensure that hospices are able to use hospice pharmacists to their greatest potential.

**Supplementary Information:**

The online version contains supplementary material available at 10.1007/s11096-021-01281-8.

## Impacts on practice

- The role of the hospice pharmacist varies depending on the time the pharmacist works within the hospice and the complexity of supply chains for that hospice.
- Hospice pharmacists do not have access to role specific training and often have limited access to patient records leading to potential impacts on patient safety.
- The involvement of pharmacists in patient care has been proved in other settings however they are yet to fulfil their potential within the hospice environment.

## Introduction

Hospices in the UK are usually independently run by charities with most funding coming from donations [1]. They care for patients with life-limiting conditions and offer symptom control, psychological and social support and bereavement care amongst other services, although each hospice has a unique offering. Most care (83%) is delivered in the community setting through home care, outpatient services and day care however, some patients are admitted for symptom control or inpatient care towards the end-of-life [1].

Pharmacists have been established members of the hospital multidisciplinary team for many years and their involvement in care has been shown to provide numerous benefits such as reduced errors, reduced readmission rates and increase in patient knowledge about medicines [2–5]. Despite this they are yet to have an established role within the hospice environment. A recent study shows 75% of UK hospices have some pharmacist provision but most of this is part-time with the pharmacist cover per bed below adequate levels [6]. In the last year of life, patients often require increasing symptom control and changes to medication in terms of dose adjustments and deprescribing of medication for long-term conditions [7, 8].

On average, a patient in the last year of life takes 11.5 medications reducing only to 10 at the point at which they die [9]. This amount of medication will inevitably lead to medication related problems (MRPs) (such as adverse drug reactions and side effects) which can be addressed by a pharmacist [10–12]. Involvement of pharmacists in the palliative care team has been shown to potentially reduce symptoms and side-effects and improve patient’s knowledge about their medicines [12–15]. Doses of medication in hospices may be higher than for patients in community settings due to severity of their illness and regimens are often complex [9]. Outpatients will usually manage medicines on their own and some may have access to community specialist palliative care nurses to help them manage their symptoms, although even patients receiving specialist palliative care can have MRPs that are yet to be addressed [11, 12].

International studies show the role of the hospice pharmacist varies between countries, however little evidence exists of good quality international comparison [16]. In Poland, hospice pharmacists were found to have little clinical input and contact with patients and the role of hospice pharmacist (HP) was non-existent in Ukraine [17]. In Canada and Australia however, pharmacists conduct medication review within the hospice and Canadian pharmacists routinely provide patient consultations focused on pain and symptom management [18]. In Canada, multidisciplinary team (MDT) working was common in the form of ward rounds and team meetings but less so in Australia and England with MDT working rare in Poland [6, 17–19]. All surveys where pharmacists were working in hospices, showed provision of information for the rest of the MDT was a common duty and administrative duties such as medicine sourcing and supply were common in the UK and Poland although less commonplace in Canada and Australia [6, 17, 18].

A number of surveys have indicated levels of hospice provision around the world but the HP role is yet to be explored [6, 17–19]. American guidelines on the essential and desirable activities of hospice pharmacists were published in 2016 but it is unclear whether these have been adopted into practice locally or worldwide [20].

### Aim of the study

The aim of this study is to explore the role of UK hospice pharmacists and to identify barriers and facilitators to the role.

### Ethics approval

The study was approved by University of Leeds Medicine Ethics Committee (MREC 18-065) in March 2019.

## Method

Purposive sampling was carried out and pharmacists working in all 17 hospices from one geographical area in the north of England invited to take part. Contact emails or telephone numbers were obtained through hospice managers, hospice administration staff and local networks. Pharmacists who responded to this initial approach were sent a participant information leaflet with details about the aims of the research and consent form, which was provided again at the time of the interview. Participants were given the choice as to where the interview would take place as long as confidentiality was maintained. A semi-structured interview schedule was constructed by the research team based on the aims of the study and included background, experience, patient contact, MDT working as well as barriers and facilitators to the role (see Supplementary electronic material). This was then piloted with a HP and refined accordingly. The target sample size was 15 or when data saturation was reached [21].

Interviews were conducted face-to-face between July 2019 and January 2020 by the lead author (ZE) who has a background in pharmacy and research. Interviews were conducted in the hospice or at the participants home (depending on their preference) and were audio-recorded and transcribed verbatim. Reflective notes were written immediately following the interview to aid analysis. The interviews were semi-structured and this technique was employed to guide the discussions yet allow unexpected findings to be explored [22]. Framework analysis was carried out to allow comparison across participants whist still maintaining individual context [23]. The steps in this analysis were data familiarisation, construction of an initial coding framework, data sorting and indexing, reviewing the data to refine the framework followed by summary of the data [23]. Transcripts were read and re-read before the framework was developed. Two researchers (ZE and SP) coded independently after which the framework was refined and then data was added to the framework. Any disagreements were resolved through discussion to reach an author consensus. A group discussion was held (with ZE, SP and EC) to agree concepts.

## Results

Of 17 hospices in the geographical region, 16 were identified as having pharmacist provision. Fifteen pharmacists (covering 14 hospices) consented to interview and two did not respond. Fourteen were interviewed in the hospice in which they worked and one in their own home. Interviews lasted between 30 and 55 min (mean 41.9, SD 8.5). Data saturation was apparent after the fifteenth interview. Demographic information is presented in Table 1 and data are categorised to protect anonymity. Thirteen participants were female and two were male. Three hospices had pharmacy services provided by more than one pharmacist. Most pharmacists were employed by hospitals, one was employed by a tertiary trust, one by a community organisation, one by a wholesaler and one by a community pharmacy. Participants worked between 1.25 and 37.5 h per week in the hospice (mean 14.3, SD 13.9). Pharmacists had been in their roles between 3.5 months and 28 years.

**Table 1:** Demographic information for hospice pharmacist participants (data are grouped together to ensure anonymity)

Participant	Age	Employer	Hospice	Hospice hours worked per week^b^	Total hospice pharmacist hours per week^b^	Length of time in role (years)	Is hospice pharmacy their only role?
HP1	25–34	Hospital	A	High	High	2 or under	Yes
HP2	45–54	Hospital	B	High	High	> 10	Yes
HP3	45–54	Hospital	C	High	High	> 10	Yes
HP4	25–34	Hospital	B & D	High	High and high	2 or under	Yes
HP5	55–64	Other^a^	E	Low	Low	> 10	No
HP6	25–34	Hospital	F	Medium	Medium	2 or under	No
HP7	35–44	Hospital	G	Low	Low	3–9	No
HP8	45–54	Hospital	G	Low	Low	> 10	No
HP9	35–44	Hospital	D	Medium	High	> 10	Yes
HP10	35–44	Hospital	H	Low	Low	3–9	No
HP11	45–54	Hospital	I & J	Medium	Medium and medium	3–9	Yes
HP12	45–54	Other^a^	K	Low	Low	2 or under	No
HP13	45–54	Other^a^	L	Low	Low	> 10	No
HP14	45–54	Hospital	M	Low	Low	> 10	No
HP15	45–54	Other^a^	N	Low	Low	2 or under	No

^a^Other represents pharmacists employed by a community organisation, wholesaler, tertiary trust of community pharmacy

^b^Respondents were categorised into one of three groups based on the number of pharmacist hours: low (2–7.5 h/week), medium (8–18.75 h/week), high (19–67.5 h/week). Categories are defined as previously described [5]

### Interview findings

Two overall themes were identified for HPs; task and communication. Ten further subthemes were found.

#### Task

The HP’s role was varied, and six sub-themes were identified (see Table 2).

**Table 2:** Qualitative responses showing the tasks of the hospice pharmacist

Task
*Medicines reconciliation*
“what I do is what I call a level 2 ….so it’s a little bit more in-depth, you’re looking at the bloods, you’re looking at all you know the doctors notes and everything rather than just a quick interactions check which is what they had really before I started… The SLA recommends we do an 80% cover." HP6 “patients in theory are meant to have a medicines reconciliation within 24 h of admission which clearly doesn’t, can’t. I mean it happens in so much as the doctors do something but …because obviously I only come once a week that we obviously don’t meet that target” HP14
*Knowledge transfer*
“I think mentoring prescribers and supporting prescribers in their decision making and giving them confidence, educating them right, informally like on the job, loads of that we do.” HP2 “And so I do a little talk about managing medicines generally and inhalers and inhaler technique, spaces, washing spaces, different kind of devices to check if your inhalers might be working if they are worried about that and then just breathlessness. But it’s quite a low level.” HP10 “I’ve not had any formal training no so it’s kind of you know… I’ve just picked it up as I go along…done a lot of reading and stuff yeah" HP11 “probably a lot of ad hoc courses…. but they are very expensive, they are sort of aimed at err medical staff erm and the continuing professional development that we get paid for at the hospital means that we barely pay for 50% of any courses that we go on so then we are having to pay for the other 50% …they’re not something you can do very often so it has actually been quite a number of years ago" HP3 “cause we’re expected to be specialists in 2 therapeutic areas….So by definition, we’re not gonna have the same expert insight….we do have a level of expertise, but, it wouldn’t be the same as somebody that works full-time in hospice." HP8
*Administrative duties*
“I focus on audits basically…. treatment/clinic room temperature, fridge temperature, emergency boxes whether they are up to date or not, whether any medication needs replacing or it’s going out of date and again needs replacing and also CD cabinet check which I’m only able to do once a month.” HP15 “[I chair meetings] making sure that we have our up to date SOPs and anything medicines related….. the [incidents reports]..looking for trends and themes, err and also ….. just sort of managing how much we’re spending on medication within the hospice.” HP1
*Undefined role*
“I think there’s quite a lot of scope for developing the role, uhm…but like it’s time-limited on, you know, if we’re only here for say, a three-hour slot, half a day…what else can we develop that’s sustainable and keep the core functions that a pharmacist needs to do? “ HP12 “My duties well, my duties are very different to what the hospice wanted then but I spoke to the management and I said look, this is not do-able…..Yes because that’s not enough time” HP15 “so [the doctor] might ring and say “I’ve got this really difficult patient at the hospice. Would you be able to pop over?”, and we would go “Yeah, alright.” HP8 “I suspect they’d just call if they had any problems.” HP7 “I am trying to implement SOPs …. Unfortunately, I’m a little bit behind because [my technician] has been off for like 3 or 4 months so I’m only working 2 ½ days it’s quite hard to get up to speed with everything but it’s all in the pipeline” HP6 “I’ve got enough time to do the minimum” HP11 “I think it’s just they’ve been more used to dealing with things in their own way here and it’s taken a long, even though I’ve been here [several years] it’s taken them that long to actually use me properly” HP11 “I wouldn’t be doing 2 parts of the process [prescribing and dispensing] so unless it’s an emergency or extreme sort of circumstances no, so that’s why the clinics [are] useful because actually if you’re prescribing in a clinic then you’re not actually dispensing because you’d be providing prescription or advice to the patient to go and get that from a registered pharmacy " HP3
*Medicines sourcing*
“for example, we’ve had a patient that’s just gone home this morning…. we [ordered their take-home medication] last week, then on Friday morning they changed, they decided to add err sodium picosulphate and then I ordered that so hopefully it was going to come on Friday afternoon, however it didn’t get sent ‘cos they didn’t have any and she was going home at 10 o’clock this morning, so I’ve had to arrange for it to be ordered on an FP10 through a local pharmacy….. quite a bit of my time is more about supplies which in some ways I feel like it takes me away from the clinical aspects because I’m just running around trying to make sure we’ve got stuff “ HP1 “You end up spending a lot of time dealing with [stock] problems and trying to sort them out and often it’s liaising with you know maybe the hospital, maybe with community team or maybe a community pharmacy as well just trying to source products or find out where they’re available, maybe manufacturers as well…..Just wasting some time “ HP3
*Person-centred care*
“not all of them are able to talk with me very well or you know they’re resting and sometimes it’s not the most appropriate to discuss with them at the time" HP4 “it’s very ad hoc, it depends on the patient and what questions they might have you know if they’re very closed and they don’t really want to know much then you probably won’t” HP3 Interviewer: “Ok, so when would you do that prescribing?” Participant: “In the MDT…..I mean, you could say that that’s dodgy, because you haven’t actually clocked the patient yourself, uhm…but if you’ve got an MDT setting, and you’ve asked all the right questions….” HP8 “Yeah it is because you don’t really know whether you need to please CQC with those audits or really be for those people" HP15 “When they go home again, I always feel like we maybe don’t provide [for them], that it would definitely be a good opportunity to give them, you know, [let them] ring up and say “I’ve come home and now it’s worse” HP9 “I think certainly it would be nice to spend more time actually sitting down with the patient and talking about the symptoms” HP14

### Medicines reconciliation

Medicines reconciliation (MR)^1^ was described by many of the participants as part of their role. Some HPs had targets of patients that should have a MR carried out by their service level agreement (SLA) even if they were part-time and there was no other pharmacist cover. Even in hospices which had high HP hours, full medicines reconciliation did not occur due to lack of around the clock provision and cover for annual leave meaning HPs not always being present when patients were admitted.

Whilst MR was structured in many hospices with targets and guidelines, some HPs simply did the medicines reconciliation if they could fit it in with little training or guidance to complete it.

### Knowledge transfer

Education of other staff, either formal or informal, was carried out by many HPs interviewed. More established or experienced HPs saw this as a long-term method of aiding development of other staff but also saw that it would be beneficial for them too; preventing errors and improving prescription quality. Education of staff was seen less in those working in low HP hours hospices, but one pharmacist did talk about education of patients, albeit at a low level.

Whilst HPs contribute to education of patients and other members of the MDT, they still have learning needs themselves. Many HPs spoke of no specific training for the role and although some had attended a two-day multidisciplinary course, this was not ideal, and barriers such as time and costs still existed. As the HP role was often only part of their overall work, some participants said it was difficult to be an “expert” in the hospice pharmacist role.

### Administrative duties

All HPs spoke of administrative duties within their roles. For those in low HP hours hospices, this was often focused on audit and was sometimes their only role within the hospice. HPs often spoke of taking the lead on investigation of error monitoring and when time allowed were involved in medicines management and other interdisciplinary meetings. Guideline and procedural writing such as Standard Operating Procedures (SOPs) were discussed as ad-hoc roles and HPs were also involved, to differing extents, in the medicine budgets for the hospice.

### Undefined role

The HP role was undefined with some HPs being unsure of what duties they should prioritise but also ideas of where they could develop the role (even though they may not have enough funding or time to do so). HPs were required to react to situations as they occurred within the hospice environment and provide assistance on demand. Many circumstances could not be planned for (for example new admissions with difficult regimens). One HP was more passive in their reactivity and assumed that team members would contact them if they needed them.

Often pharmacists were the only one in their role in the hospice so when issues such as holidays or absences occurred, a level of resilience was needed. Part-time working was common with over half of the participants working 7.5 h a week or less within the hospice environment. This led to problems in service delivery and continuity.

Change seemed hard to achieve within the hospice environment and often HPs carried on with similar duties from their predecessors. HP time was often utilised by duties that were not necessarily, in their opinion, the best use of their time.

HPs did talk about what difference a prescribing qualification would bring to their role. One HP was qualified but did not use it, and two were, at the time of the interviews, training for the qualification. Issues such as safety (if they were writing the prescription and dispensing it) and confidence for HPs in medium or low HP hours hospices were also discussed. Some HPs thought the prescribing qualification would be useful but there was also some doubt as to whether that would force them to sacrifice other duties within their workload.

### Medicine sourcing

All hospices had slightly different methods of sourcing medicines. Current issues with medicines shortages and complex delivery pathways often impacted on the HPs working day where they would have to spend time trying to source medication for patients. One hospice obtained their medicines from the local hospital via a tertiary hospital and had one delivery a day. This could lead to problems when patients had rapidly changing conditions. Changes to UK medication licencing in recent years has added to the costs and complexity of hospice medication supply making once simple supply chains more convoluted for many hospices..

Current shortages are not always well communicated due to different models operating in hospices, hospitals and community pharmacies. This can lead to extra time being spent on obtaining patient medication.

### Person-centred care

HPs displayed person-centred care and compassion in the way they spoke about their role. They talked about proactively tailoring care to the individual needs of the patient by crushing tablets when the patient could not swallow or organising dosette boxes for patient to go home with. Sometimes care was person-centred in the decision where a seriously ill patient was not disturbed to discuss their medication. Care was sometimes delivered in what could be considered, less person-centred ways by prescribing for a patient the HP had never met.

There was confusion as to how to do the right thing for patients whilst also working towards the priorities of managers and hospice targets. One HP saw a gap in how care could be delivered in a more person-centred way by providing remote services for when patients have been discharged. When asked what they would do if they had more time in their roles, HPs saw person-centred care as a priority.

#### Communication

Communication was a common theme, and this is split into the different forms of communication HPs were involved with (see Table 3).

**Table 3:** Qualitative responses showing communication of hospice pharmacists

Communication
*Routine*
“I try to go on them [ward rounds], it will depend on the timing, how long they go for but if it is quite busy I will try and hang around in the office so I’m there for sort of the pre and post discussion of ward rounds… that could go [on] for 4 h” HP3 “[the] best [thing] about being in [this hospice], cause [the doctors] always come back in and just will discuss, and you’re just there so, you kind of absorb all this information, just by being there…” HP9 [talking about sharing an office with the doctors] “I’m coming to do the ward round then……unpaid, yes.….I feel like it does help me do my round checking charts, I can actually envisage the patient and what’s going on with them a bit better if I’ve been on the ward round.” HP10
*Ad-hoc*
“we carry a phone so the doctors can contact us erm so we’re very much involved in the day to day medicines decision making…. it’s very much part of the MDT" HP2 “As I said if I’m in the ward office, they will just come over and ask…., on the inpatient unit people will come and knock at the door ….but I think it depends on the experience of the staff and what other staff are about …. Sometimes the consultants might come in and talk about symptoms but probably less so. HP3
*Electronic/information sharing*
“I very rarely get to see all the meds that they’ve come in on so it’s more a case of trying to work out what’s been going on and looking on SystmOne….if they’ve got any notes, if they’ve come over from hospital I can go through the notes from hospital but sometimes it’s difficult to get a true record of what patients have been on…. I’ve only got nurse role [on the electronic system] which is a bit frustrating” HP11 “you get patients say here erm who have the list of medications from the GP but that isn’t kept up to date with any changes that are made by other teams and you get people who are sort of on a drug that is never provided by the GP and the GP doesn’t actually include that on the records” HP13 “it’s a case of piecing everything together you know …. I think things are not terribly joined up because everyone’s using different systems I think that’s the problem so you are relying on the relevant letters being printed out or received." HP14
*Networks*
“so sometimes [the doctors] want to start a blister pack, so I might ring the chemist and check, you know, that that was alright, discuss that with them and… depending on the community pharmacy, obviously, as to how much they know about the patients… and they’ll say “We’ve tried that in the past” or”It hasn’t worked” cause everyone thinks blister packs are amazing but, actually for our patients, you’ve still gotta pop them out" HP9 “cause I’ve been here so long, I guess I know most of the community nurses anyway, cause we try and train them when they’re doing their prescribing….I guess that’s a good thing about being here so long, isn’t it? They’re…kind of all aware.” HP9

### Routine

Regular communication with the rest of the MDT could occur in several ways. The MDT team meetings and ward rounds were both opportunities, although these had the potential to take up a lot of time and did not necessarily correspond with the working days and hours of the HP, particularly with low and medium hours pharmacists. The meetings were not always perceived to be a good use of time and the benefits of this had to be weighed up against other priorities. Alternative ways of routine communication such as daily handovers and post ward round discussions were found to be useful by some participants.

Some HPs found this routine communication to be a form of bouncing ideas around the MDT and it was recognised that it may be beneficial to attend if they had additional hours within the hospice. One HP thought that the benefit of them attending the ward round was so great they opted to do it in their own, unpaid time.

### Ad-hoc

HPs were often required to react (as seen in undefined) but they are also often required to communicate in a reactive way. This involved answering queries from the MDT and this could be by phone or one of the MDT coming and finding them in-person. This was seen more often from less experienced members of staff and there was a need to fit this in alongside other duties and prioritise accordingly.

### Electronic/information sharing

HPs require information about the patient to safely carry out clinical duties. This includes current and previous medication, diagnosis history and biochemistry. In some settings these are carried electronically, some are paper-based and some are a combination. HPs had a mixture of access to patient information. Often it was time-consuming to find the relevant information about the patient and it was not clear whether the full picture had been obtained. The lack of access to medical records may be due to system failures as primary, secondary and tertiary care use separate systems. Often systems which were available were incomplete and did not contain records from other settings.

### Networks

HPs often use networks of healthcare professionals to improve patient care. This may be by liaising with community pharmacists regarding individual patient needs at discharge regarding obtaining or dispensing medication.

Networks had also been created when the HP had been in their role for an extended period of time. This often meant that they know who to contact for which issue and this may make their job easier. HPs who had limited time in the hospice or were not established in the hospice may find it difficult to develop the networks they need to provide the best care.

## Discussion

This study explored the role of the hospice pharmacist in the UK. The findings show the varied tasks of hospice pharmacists and the attributes useful for the role. Challenges were shown in communication, knowledge, sourcing medicines and general time pressures with most HPs working part-time. This is the first study to qualitatively explore the role of a HP, although some surveys have been conducted concerning duties, hours and MDT working [6, 17–19]. Our main findings concur with a previous study in 1995 of HPs performing administrative, clinical, educational and supply however the role of the pharmacist now encompasses much more in terms of clinical duties than was expected 25 years ago [25].

Lack of time was an issue that was talked about frequently in the interviews as has been found elsewhere [18]. Eleven of the fifteen HPs interviewed worked less than 19 h a week in the hospice, which is similar to the results of a 2020 survey of UK hospice pharmacists showing a mean HP working week of 13 h [6]. HPs talked of scope for more person-centred services if they had time, and where time was less of a problem, they had a more integral role in training and development of other staff. Surveys in Poland, Australia and Canada showed part-time working to be by far the most common model of HP provision [17, 18]. Administrative duties were common and those working less hours often described more of an administrative role with audit and error reporting. HP prescribing in the hospice environment was being considered although may only be useful and safe for those working longer hours.

Lack of time was further adversely affected by current medicines shortages and time spent sourcing medicines. Issues have been compounded by sometimes complex sourcing pathways which may have been brought about by licencing changes in the UK [26]. Although hospices are usually funded by charities, medicine budgets are provided by the NHS meaning expensive government licences to obtain supplies from local hospitals are being paid for out of NHS budgets. The complex supply chains often involved in obtaining medicines for hospices is an unnecessary drain on resources. Although UK HPs have a major role in medicines sourcing, this is not the same in all countries [16, 18]. Time taken in sorting out issues was sometimes negated by HPs who had been in their role for a long time or were experienced in the field as they could use their networks to achieve what they needed to more quickly.

HPs showed person-centredness in many ways, from unpaid working to provide a more complete service to tailoring the service provided to the individual needs of the patient. Poland relies more heavily on unpaid hospice pharmacist time although they rarely have direct involvement in patient care [17]. In a recent review, HPs were found to have a more person-centred role in USA, UK, Australia and Canada compared with the rest of the world [16]. Person-centred care for patients at the end of life has been integrated into guidelines relating to care for this specific patient group [27, 28]. Although most HPs demonstrated this, some had different ideas about how to best use their time to improve patient care.

HPs in high HP hours hospices provided support to the wider MDT in the form of training and mentoring as seen elsewhere [16, 18]. Patient consultations were rarely described, in contrast with practice in Canada, although this may be due to differences in terminology as when carried out on admission this may be referred to as medicines reconciliation [18]. Those in lower HP hours hospices also added value in terms of medicine rationalisation and medicines specific knowledge however no HP is able to access pharmacist-specific palliative care training as available training is all multidisciplinary [6]. Lack of pharmacists with relevant skills has been found to be an issue elsewhere and other barriers identified to HP training include cost and time [18]. The HP role is yet to be defined and often participants had to work out how best to use the time they had in the hospice to benefit patients. Although service level agreements were in place in some hospices, there would be benefit from defined role objectives which were achievable and evidence-based. Guidelines on essential and desirable activities of HPs from the USA show some correlation with these findings, although the varied nature of time and training lead to large inconsistencies in practice [18, 20].

Communication of information was a key facilitator to the HP’s role. HPs had often developed methods of regular and ad-hoc communication with members of the MDT (depending on how much time they had) to pass-on or receive relevant information. The provision of information to the MDT was found to be commonplace as has been found elsewhere [16–19]. Lack of full access to patient records leads to time wasted piecing together information from different sources. A recent survey showed that only 12% of hospice pharmacists had full read and write access to the patient’s medical records which could have potential safety implications when a complete patient history is important for medicines reconciliation to be effective [6, 29].

The results of this study have led to the development of a list of barriers and facilitators for the role of HP. Barriers include lack of time, role-specific training, definition of role, complex supply chains and lack of access to full patient records. Facilitators would therefore include investment in HP time, training, role definition, efficiencies in supply chains and access to full patient records. These facilitators, if addressed may positively influence the role the HP plays within the hospice allowing the HP to help fulfil their potential as an integral member of the MDT. The facilitators will each require further research to quantify and explore their individual components.

### Strengths and limitations

This study was reliable as it could be replicated easily, and it is likely that consistent results would be obtained. Although the study was carried out in one geographical region of the UK, this was a large area with diverse populations and healthcare systems making it likely to have transferability. Although one researcher was responsible for conducting the interviews, researcher bias was addressed by the inclusion of other members of the research team in the analysis.

Interviews could be carried out on a national scale to confirm transferability and an international scale to identify practice elsewhere. Further research into effective and efficient hospice medicines supply is required. The UK would benefit from research to develop models for low, medium and high HP hours hospices to develop role guidance to ensure optimum use of resources.

## Conclusion

Although pharmacists have been present in hospices for decades, it is only now becoming apparent, the added value they could provide through their clinical expertise in medicines. HPs provide varying duties and this is often dependent on the time available in the role or the duties that have been historically carried out by the HP. Investment in training and definition of roles along with improvement of supply chains and communication systems will facilitate HPs to fulfil their true worth to hospice care at the end of life.

## Supplementary Information

Below is the link to the electronic supplementary material.

Supplementary file1 (DOCX 16 KB)

## Footnotes

1 Medicines reconciliation can be defined as “the process of accurately listing a person’s medicines. This could be when they are admitted into a service or when their treatment changes” Care Quality Commission [24]Care-Quality-Commission. "Medicines Reconciliation." CQC. https://www.cqc.org.uk/guidance-providers/adult-social-care/medicines-reconciliation-how-check-you-have-right-medicines (accessed 11th November, 2020).

## References

[1] Hospice UK. Facts and figures-about hospice care. Hospice UK. 2019. https://www.hospiceuk.org/about-hospice-care/media-centre/facts-and-figures. Accessed 13th Jan 2021.

[2] Cavanaugh JJ, Lindsey KN, Shilliday BB, Ratner SP (2015). Pharmacist-coordinated multidisciplinary hospital follow-up visits improve patient outcomes. J Manag Care Spec Pharm.

[3] Hussainy SY, Box M, Scholes S (2011). Piloting the role of a pharmacist in a community palliative care multidisciplinary team: an Australian experience. BMC Palliat Care.

[4] Kolovetsios MY, Hawre Y (2018). The role and impact of pharmacists within a hospice's care home support team. BMJ Support Palliat Care.

[5] Magedanz L, Silliprandi EM, Dos Santos RP (2012). Impact of the pharmacist on a multidisciplinary team in an antimicrobial stewardship program: a quasi-experimental study. Int J Clin Pharm.

[6] Edwards Z, Mulvey M, Chapman EJ, Bennett MI (2021). A national survey of hospice pharmacists and a comparison with international models. Int J Pharm Pract.

[7] Todd A, Husband A, Andrew I, Pearson SA, Lindsey L, Holmes H (2017). Inappropriate prescribing of preventative medication in patients with life-limiting illness: a systematic review. BMJ Support Palliat Care.

[8] Radbruch L, Hoffmann-Menzel H, Kern M, Rolke R (2012). Hospice pharmaceutical care: the care for the dying. Eur J Hosp Pharm.

[9] McNeil MJ, Kamal AH, Kutner JS, Ritchie CS, Abernethy AP (2016). The Burden of polypharmacy in patients near the end of life. J Pain Symptom Manag.

[10] LeBlanc TW, McNeil MJ, Kamal AH, Currow DC, Abernethy AP (2015). Polypharmacy in patients with advanced cancer and the role of medication discontinuation. Lancet Oncol.

[11] Pharmaceutical Care Network Europe Foundation. Classification for drug related problems V8.02. 2017. https://www.pcne.org/upload/files/230_PCNE_classification_V8-02.pdf. Accessed 2nd May 2021.

[12] Edwards Z, Bennett MI, Blenkinsopp A (2019). A community pharmacist medicines optimisation service for patients with advanced cancer pain: a proof of concept study. Int J Clin Pharm.

[13] Wilson S, Wahler R, Brown J, Doloresco F, Monte SV (2011). Impact of pharmacist intervention on clinical outcomes in the palliative care setting. Am J Hosp Palliat Care.

[14] Edwards Z, Ziegler L, Craigs C, Blenkinsopp A, Bennett MI (2019). Pharmacist educational interventions for cancer pain management: a systematic review and meta-analysis. Int J Pharm Pract.

[15] Walker KA (2010). Role of the pharmacist in palliative care. Prog Palliat Care.

[16] Krzyzaniak NP, Pawlowska I, Bajorek B (2017). An overview of pharmacist roles in palliative care: a worldwide comparison. Palliat Med Pract.

[17] Pawlowska I, Pawlowski L, Lichodziejewska-Niemierko M (2016). The role of a pharmacist in a hospice: a nationwide survey among hospice directors, pharmacists and physicians. Eur J Hosp Pharm.

[18] Gilbar P, Stefaniuk K (2002). The role of the pharmacist in palliative care: results of a survey conducted in Australia and Canada. J Palliat Care.

[19] Prokip S, Pawlowska I, Hromovyk B, Pawlowski L (2014). Pharmacist's role in the system of palliative and hospice care in Ukraine and Poland. J Med Sci.

[20] Herndon CM, Nee D, Atayee RS, Craig DS, Lehn J, Moore P (2016). ASHP guidelines on the pharmacist's role in palliative and hospice care. Am J Health Syst Pharm.

[21] Fusch P, Lawrence R (2015). Are we there yet? Data saturation in qualitative research. Qualiat Rep.

[22] Gale N, Heath G, Cameron E, Rashid S, Redwood S (2013). Using the framework method for the analysis of qualitative data in multidiciplinary health research. BMC Med Res Methodol.

[23] Ritchie J, Lewis J (2003). Qualitative research practice—a guide for social science students and researchers.

[24] Care-Quality-Commission. Medicines Reconciliation. CQC. 2020. https://www.cqc.org.uk/guidance-providers/adult-social-care/medicines-reconciliation-how-check-you-have-right-medicines. Accessed 11th Nov 2020.

[25] Lipman AG, Berry JI (1995). Pharmaceutical care of terminally ill patients. Journal of Pharmaceutical Care in Pain & Symptom Control.

[26] Rushton D. UK Home Office-Controlled Drugs Licence Applications. 2021. https://paradigmshiftconsulting.co.uk/home-office-controlled-drugs-licence/. Accessed 12th Jan 2021.

[27] National Institute of Clinical Excellence. New guidelines to improve care for people at the end of life. NICE. 2019. https://www.nice.org.uk/news/article/new-guidelines-to-improve-care-for-people-at-the-end-of-life. Accessed 21st Mar 2019.

[28] NHS. The NHS Long Term Plan. NHS. 2019. https://www.longtermplan.nhs.uk/wp-content/uploads/2019/01/nhs-long-term-plan.pdf. Accessed 21st Dec 2019.

[29] Moore P, Armitage G, Wright J, Dobranski S, Ansari N (2011). Medicines reconciliation using a shared electronic health care record. J Patient Saf.

