# Supplementary material for: Understanding the role of hospice pharmacists: a qualitative study
Source: Int J Clin Pharm. 2021 Jun 13;43(6):1546–54. doi: 10.1007/s11096-021-01281-8 (PMC8642336; doi:10.1007/s11096-021-01281-8)
Supplement: Supplementary file 1 — Supplementary file1 (DOCX 16 KB) [file 11096_2021_1281_MOESM1_ESM.docx]

APPENDIX 1

**RESOLVE Interview topic guide for Hospice pharmacists**

We have been funded by Yorkshire Cancer Research to develop interventions to benefit patients with advanced cancer who have pain, breathlessness and/or fatigue. We would like to investigate the possibility of hospice pharmacists providing telephone-based consultations for outpatients towards the end-of-life. To do this we need to find out a bit about hospice pharmacists and what might be barriers and facilitators for this to happen.

| **Theme** | **General Questions** | **Prompt items** | **Asked** |
| --- | --- | --- | --- |
| Background | - Please could you tell us your background and experience | - University - Pre-reg - Qualifications - Prescriber? - Work experience |  |
| Background | - What specific training and experience have you had in palliative care/cancer/end-of-life? | - Courses - Qualifications - Personal |  |
| Other relevant experience | - Do you have any other jobs? | - Locuming – hospital/community - Practice pharmacy - Something else |  |
| Current role | - Tell me about the role you are currently in? | - Job title - How long in post? - What duties are expected/performed? - Hours worked a week |  |
| Current role | - What contact do you have with patients? | - Ward rounds - Clinics - Ad-hoc questions from staff? - Inpatients/outpatients? - Any patient-facing review/discussion about medicines and symptoms |  |
| Current role | - How do you work with other clinicians to address pain, breathlessness or fatigue? | - Do you deal with symptoms yourself or refer – if so, to whom? - If you deal with symptoms – what evidence sources/evidence are used (by you or by other clinicians)? - Do you take part in hospice multi-disciplinary meetings/case discussions? - Would this be different if you were a prescriber? Or is it different because you are? - Do you use any patient resources? E.g. leaflets, books, websites - How is any contact made with primary care or community pharmacies? |  |
| Current role | - What other duties do you have that we haven’t talked about? |  |  |
| Services experience | - What experience do you have of providing services? | - Patient consultation skills - MUR/NMS |  |
| Facilitators | - We are specifically focusing our work on pain, breathlessness and fatigue. Do you have any experience of giving advice for these symptoms? If so, what suggestions do you have that we could share with other pharmacists. | - Pain - Breathlessness - Fatigue |  |
| Barriers | - How would you feel about doing a telephone-based medicines review with advice about pain, breathlessness or fatigue? Other than time, would you have any concerns. | - Learning needs – pain, breathlessness, fatigue? - Lack of other skills? - What other training could be provided? - Time - Access to patient records - In what form would training needs be best addressed? E.g. computer learning, paper-based, training session? |  |
